# Terminally Differentiated CD4^+^ T Cells Promote Myocardial Inflammaging

**DOI:** 10.3389/fimmu.2021.584538

**Published:** 2021-02-19

**Authors:** Murilo Delgobo, Margarete Heinrichs, Nils Hapke, DiyaaElDin Ashour, Marc Appel, Mugdha Srivastava, Tobias Heckel, Ioakim Spyridopoulos, Ulrich Hofmann, Stefan Frantz, Gustavo Campos Ramos

**Affiliations:** ^1^ Comprehensive Heart Failure Centre, University Hospital Würzburg, Würzburg, Germany; ^2^ Department of Internal Medicine I, University Hospital Würzburg, Würzburg, Germany; ^3^ Core Unit Systems Medicine, University Hospital Würzburg, Würzburg, Germany; ^4^ Freeman Hospital, Department of Cardiology, Newcastle upon Tyne, United Kingdom; ^5^ Translational and Clinical Research Institute, Cardiovascular Biology and Medicine, Newcastle University, Newcastle upon Tyne, United Kingdom

**Keywords:** CD4+ T-cells, myocardial aging, inflammaging, NSG animals, immunosenescence, lymphocytes

## Abstract

The cardiovascular and immune systems undergo profound and intertwined alterations with aging. Recent studies have reported that an accumulation of memory and terminally differentiated T cells in elderly subjects can fuel myocardial aging and boost the progression of heart diseases. Nevertheless, it remains unclear whether the immunological senescence profile is sufficient to cause age-related cardiac deterioration or merely acts as an amplifier of previous tissue-intrinsic damage. Herein, we sought to decompose the causality in this cardio-immune crosstalk by studying young mice harboring a senescent-like expanded CD4^+^ T cell compartment. Thus, immunodeficient NSG-DR1 mice expressing HLA-DRB1*01:01 were transplanted with human CD4^+^ T cells purified from matching donors that rapidly engrafted and expanded in the recipients without causing xenograft reactions. In the donor subjects, the CD4^+^ T cell compartment was primarily composed of naïve cells defined as CCR7^+^CD45RO^-^. However, when transplanted into young lymphocyte-deficient mice, CD4^+^ T cells underwent homeostatic expansion, upregulated expression of PD-1 receptor and strongly shifted towards effector/memory (CCR7^-^ CD45RO^+^) and terminally-differentiated phenotypes (CCR7^-^CD45RO^-^), as typically seen in elderly. Differentiated CD4^+^ T cells also infiltrated the myocardium of recipient mice at comparable levels to what is observed during physiological aging. In addition, young mice harboring an expanded CD4^+^ T cell compartment showed increased numbers of infiltrating monocytes, macrophages and dendritic cells in the heart. Bulk mRNA sequencing analyses further confirmed that expanding T-cells promote myocardial inflammaging, marked by a distinct age-related transcriptomic signature. Altogether, these data indicate that exaggerated CD4^+^ T-cell expansion and differentiation, a hallmark of the aging immune system, is sufficient to promote myocardial alterations compatible with inflammaging in juvenile healthy mice.

## Introduction

Immunosenescence is a conserved phenomenon among a wide range of animals, including birds, reptiles and mammals, which strongly correlates with morbidity in elderly humans (1, 2). Senescence particularly manifests in adaptive immune functions, as evidenced by a reduction in naïve T cell production, an accumulation of effector and terminally differentiated T cells and a decline in antibody response following antigen stimulation in aged individuals (3, 4). In parallel to these alterations, chronic, sterile, low-grade inflammation also develops alongside aging, a process termed inflammaging, which contributes to the pathogenesis of a multitude of age-related diseases (5). T cell dysfunction during aging stands at the crossroad between immunosenescence and inflammaging. Effector memory T cells (T_EM_) (CD44^+^CD62L^-^ in mice; CD45RO^+^CCR7^-^ in humans) may develop a senescent phenotype characterized by the expression of checkpoint inhibitory receptors and defective TCR-mediated proliferation, but increased secretion of proinflammatory cytokines (6). These paradoxical conditions result in immunodeficiency and inflammation, dampening anti-infectious responses while fostering tissue damage and autoimmunity (7). Mechanistically, T_EM_ dominates the aged immune system compartment due to a couple of factors, including thymus involution and long-life exposure to pathogenic stimuli. In particular, immune surveillance against persistent viruses, mostly cytomegalovirus (CMV), causes an inflation of the effector/memory T cell compartment and an early onset of the immune aging phenotype in humans (3, 8).

In light of the profound effects of immune aging in human health, recent studies sought to discover biomarkers for physiological and accelerated aging. Early data from the OCTO-immune longitudinal study revealed that octogenarians with a more advanced immunosenescence profile, characterized by a high frequency of CD8 over CD4 lymphocytes and poor proliferative responses to mitogen stimuli, also presented higher mortality (9). The follow-up NONA-immune longitudinal study further confirmed the positive association between a high immune risk profile and higher mortality in nonagenarians. In addition, higher levels of IL-6 were associated with poorer cognitive function and predicted future cognitive decline in the elderly (10). Most recently, longitudinal high-dimensional immune profiling of young and elderly humans generated an immune aging (IMM-AGE) score, which could better indicate immunological aging, regardless of chronological factors. At the cellular level, interindividual shifts in CD4^+^ and CD8^+^ T cell subsets accounted for the immune trajectories observed in aging. By selecting a set of transcripts and surface markers that represent such immune aging trajectories, the authors showed that the IMM-Age score could better predict cardiovascular disease and overall mortality than epigenetic clock markers (2).

Compelling clinical and preclinical data have now demonstrated that exaggerated immunosenescence profiles are associated with several aging-related diseases in humans. In particular, T cell dysfunction during aging has also been associated with rheumatoid arthritis, systemic lupus erythematosus, metabolic disorders, and more specifically, cardiovascular diseases. The senescent CD4^+^ T cell compartment has been implicated in myocardial inflammation and has been shown to promote age-related tissue dysfunction. Aged T cells from heart-draining lymph nodes show preferential heart homing and a proinflammatory response in young transferred mice (11). In humans, T cell senescence due to CMV infection predicts cardiovascular mortality in the elderly population (12). Moreover, Moro-Garcia et al. found that CMV seropositivity and its ensuing T cell memory inflation correlated with more advanced heart failure progression in middle-aged patients (12, 13). In addition, CMV-independent maladaptive accumulation of CD28^-^ T cells positively correlates with cardiovascular death, whereas memory CD8^+^CD28^+^ T cell numbers are associated with overall improved survival (14).

Taken together, these lines of evidence suggest that an accumulation of a senescent T cell compartment may contribute to age-related myocardial decline and predispose toward cardiovascular diseases. However, it remains unclear whether aged T cells would simply fuel cardiac-intrinsic age-related damage or act as primary triggers of myocardial aging even in the absence of other preexisting conditions. To mechanistically decompose this question, we generated a xenograft transplantation model in which young humanized mice were treated to harbor a senescent-like expanding human CD4^+^ T cell compartment. Our data uncover a role for terminally differentiated T cells in promoting cardiac inflammatory shifts that match those observed in aged hearts.

## Materials and Methods

### Study Approval

For mouse studies, all *in vivo* procedures were approved by the local authorities (*Regierung von Unterfranken*) and conformed to the guidelines from Directive 2010/63/EU of the European Parliament on the protection of animals used for scientific purposes.

The human samples were obtained from another study conducted at the university clinic of Würzburg that had previously recruited 46 patients with ST-elevation and non-ST-elevation infarction (STEMI/NSTEMI), 48 healthy control patients who had undergone elective coronary angiography without any sign of coronary artery disease, and 15 healthy volunteers (KAMI-study). Individuals with active tumors, hematological diseases, skeletal muscle diseases, autoimmune diseases, immunosuppressive treatment, or infection were excluded from the study. All parts of the study conform to the Declaration of Helsinki and have been approved by the respective ethics committee of the University of Würzburg. All patients signed an informed consent form.

### Animals

Adult (2–3 months) and aged (12–16 months) C57/BL6J mice were purchased from Charles River, whereas 2–3-month-old NOD-Prkdc^Scid^ Il2rg^tm1Wjl^ H2-Ab1^tm1Doi^ HLA-DRA*0101 and HLA-DRB1*0101 (NSG-DR1) were commercially available from Jackson Laboratory though a dedicated-breeding project (stock # 030331). Due to the combined *Prkdc* ^Scid^ and *Il2rg* ^tm1Wjl^ mutations, these animals harbor no mature T or B cells, lack functional NK cells, and are deficient in cytokine signaling, making xeno-engraftment possible without signs of rejection (15). Moreover, these animals express a transgenic construct containing the mouse MHC Class II *H2-Ea* and *H2-Eb1* genes engineered to encode proteins in which the alpha1 domain (amino acids 1-85) and the beta1 domain (amino acids 1-96) were replaced by the corresponding amino acids of the human MHC Class II protein encoded by HLA-DRA1*0101 and HLA-DRB1*0101. This mutation allows human MHC-II molecules to be functionally expressed on murine antigen presenting cells, which could then prime engrafted human CD4^+^ T cells.

The animals were housed under specific pathogen-free (SPF) conditions with a 12-h light/12-h dark cycle and standard diet provided ad libitum and were acclimatized for at least 7 days post shipment. Due to their immunodeficiency status, the NSG-DR1 mice were maintained in dedicated ventilated cabinets, and all experimental procedures were performed inside a laminar flow hood under sterile conditions.

Human peripheral blood samples were obtained from healthy subjects recruited into the aforementioned clinical study in our hospital (Department of Internal Medicine of the University Hospital of Würzburg) in a period ranging between 2018 and 2020. Blood samples were collected in CPT vacutainers coated with sodium heparin and Ficoll^TM^ Hypaque^TM^ (BD, Franklin Lakes, USA), and peripheral blood mononuclear cells (PBMCs) were obtained after gradient centrifugation (1500 *g*, 20 min, 23°C), frozen in CTL-Cryo^TM^ freezing media (CTL, Boon, Germany) and then stored in liquid nitrogen (-180°C) until further usage. To monitor the effects of aging on the peripheral blood T cell compartment, we performed eight color flow cytometry measurements on all healthy subjects who had been recruited to the aforementioned study (described below). In total, we analyzed 13 samples from young subjects (20–45 yo), 18 samples from middle-aged subjects (45–65 yo) and 26 samples from elderly subjects (65–85 yo). A description of these subjects is presented in **Supplemental Table I**.

HLA genotyping was performed by the Institute for Clinical Transfusion Medicine and Haemotherapy from the University Hospital of Würzburg as previously described (16), and only CD4^+^ T cells from matching healthy donors (i.e., DRB1*01:01) were selected for adoptive transfer experiments. Out of 57 healthy subjects screened, we found seven matching samples suitable for transplantation into NSG-DR1 mice. Three of these subjects had undergone elective coronary angiography evaluations with excluded myocardial infarction, and the remaining subjects were healthy volunteers. A description of the healthy donors enrolled in the T cell transfer experiments is presented in **Supplemental Table II**.

### Adoptive Cell Transfer

Human untouched CD4^+^ T cells were purified from PBMC samples by magnetic cell sorting using the CD4^+^ T cell isolation kit, Human (Miltenyi Biotec, Bergisch Gladbach, Germany). Finally, human CD4^+^ T cells were resuspended in sterile PBS (Biochrom, Berlin, Germany) at a concentration of 1x10^7^ cells per ml and adoptively transferred into NSG-DR1 mice (2x10^6^ cells i.p. – referred to as day 0). Baseline characterization of the donor cells and purity checks were routinely performed by flow cytometry. As a control group, age-matched naïve NSG-DR1 littermates were investigated.

### Endpoint Analysis

At 6 weeks post human CD4^+^ T cell transfer, NSG-DR1 mice were sacrificed by cervical dislocation, then whole-body perfusion with PBS + 50 U/ml heparin (Ratiopharm, Ulm, Germany) was conducted and the organs were extracted for *ex vivo* analysis. FACS analysis of freshly isolated cells (heart and spleen) was performed. The samples intended for RNA analysis were stored in RNAlater (Qiagen, Hilden, Germany) for 24 h and then stored at -80°C. Samples intended for histological analysis were embedded in Tissue-TeK optimum cutting temperature medium (Sakura Finetek, Alphen ann den Rijn, The Netherlands) and then stored at -80°C. Heart DNA extraction was performed from heart slices by trimming the Tissue-TeK content and performing tissue digestion and DNA purification using a GeneJET genomic DNA purification kit (Thermo Scientific, Vilnius, Lithuania) according to the manufacturer’s instructions.

### *In Vitro* Stimulation Assay

1 million PBMC (pretransfer), NSG-DR1 recipients’ splenocytes or digested heart tissues were resuspended in complete RPMI media containing 10% FCS, 1% L-Glutamine, 1% Sodium pyruvate, 1% non-essential amino acids, 1%Pen/Strep, 1 µM 2-ME (Gibco – Grovemont Cir, USA) and seeded in U-bottom 96-well plate. Cells were stimulated for 3 h with a T-cell stimulation cocktail containing Phorbol 12-Myristat 13-Acetat (81 ηM) und Ionomycin (1.34 µM) supplemented with protein transport inhibitors Brefeldin A (10.6 µM) and Monensin (2µM) (eBioscience – San Diego, USA). Following incubation time, cells were washed and used in intracellular flow cytometry analysis.

### Flow Cytometry

Immunophenotyping of spleen and digested heart samples obtained from control and CD4^+^ T cell transfer mice and human PBMCs was performed. The heart samples were enzymatically digested in type II collagenase (1,000 IU/ml, Worthington Biochemical Corporation, Lakewood, NJ, USA) for 30 min at 37°C and then ground against a 70-µm mesh (Miltenyi Biotec, Bergisch Gladbach, Germany) in 0.5% BSS/BSA. The lymphoid organs were ground against a 30-µm cell strainer, and the splenocyte preparations underwent erythrocyte lysis. All samples were stained with zombie aqua fixable viability dye (BioLegend, San Diego, USA) for 15 min at room temperature and protected from light. Afterwards, samples were washed and resuspended in FACS buffer, and surface staining was performed in the presence of FC-blocking antibody (anti-CD16/CD32, clone 2.4G2, BD Pharmingen) for host NSG-DR1 cell panels. The following antibodies conjugated with different fluorophores were used:

Human: anti-CD45RO (clone UCHL1), anti-CD4 (OKT4), anti-CCR7 (clone G043H7), anti-CD25 (clone M-A251)anti-CD127 (clone A010D5) obtained from BioLegend (San Diego, USA), and anti- PD-1 (clone J105), anti-FoxP3 (clone 236A/E7), anti-TNF (clone Mab11), anti-IFN-γ (clone 45.B3), anti-IL-17a (clone eBio64DEC17) and anti-IL-13 (clone 85BRD) obtained from eBioscience (San Diego, USA). Mouse: anti-CD45 (clone 30-F11), anti-CD11b (clone M1/70), anti-Ly6G (clone 1A8), anti-CD11c (clone N418), anti-CD62L (MEL-14), anti-CD3e (145-2C11), anti-CD44 (IM7), and anti-CD4 (RM4-5) conjugated with different fluorophores were obtained from BioLegend (San Diego, USA). Flow cytometry measurements were performed on an Attune-NxT (Thermo Scientific, Darmstadt, Germany). Data analysis was performed using FlowJo software (FlowJo LLC Ashland, OR, USA). Compensation for spectral overlap was conducted based on single staining controls, and flow cytometry gates were set based on unstained controls and fluorescence minus one controls.

### Gene Expression Analysis

RNA was extracted from mouse myocardial samples (apical region) using the tissue RNA isolation kit (RNeasy mini – Qiagen). The RNA concentration and quality were assessed in a spectrophotometer, and 200 ng was used for cDNA synthesis (iScript – Bio-Rad). TaqMan probes for quantitative PCR were used to measure the expression of genes related to cardiac stress, aging, inflammation and fibrosis. The probes used in this manuscript were *Gapdh* (Mm033002249_g1), *Hsp1a1* (Mm01159846_s1), *Myh6* (Mm00440359_m1), *Myh7* (Mm01319006_g1), *Tnf* (Mm99999068_m1), *Il1b* (Mm00434228_m1), *Mmp9* (Mm00442991_m1), *Mmp2* (Mm00439498_m1), *Col1a1* (Mm00801666_g1), *Col3a1* (Mm01254476_m1), and *Tgfb3* (Mm00436960_m1). Target mRNA levels were normalized to *Gapdh* expression levels.

### RNA-Seq and Bioinformatics

RNA extracted from mouse myocardial samples (apical region) with or without human T-cell transfer were used for bulk RNA sequencing. DNA libraries suitable for sequencing were prepared from 220 ng from one control sample and from 400 ng of total RNA from all other samples with oligo-dT capture beads for poly-A-mRNA enrichment using the TruSeq Stranded mRNA Library Preparation Kit (Illumina). Sequencing was performed on the NextSeq-500 platform (Illumina) in paired-end mode with 2x75 nt read length. Sequencing data are available at NCBI GEO (http://www.ncbi.nlm.nih.gov/geo) under the accession number GSE163413. Sequencing reads were adapter trimmed and mapped to the mouse (GRCm38.p6) and human genome (GRCh38.p13) with STAR followed by computational deconvolution of mouse and human reads using the XenofilteR tool (17). Gene level based read counts were generated with featureCounts using the RefSeq annotation. The count output was utilized to identify differentially expressed genes using DESeq2. Gene ontology (GO) and Kyoto Encyclopedia of Genes and Genomes (KEGG) orthology analyses were performed using the web server g:Profiler (18) for all upregulated transcripts (for mouse transcripts: log2 fold change >0.5, FDR<0.05). The upregulated transcripts were also analyzed against the myocardial single cell atlas derived from *Tabula muris senis* consortium (19). Briefly, the gene counts and metadata from heart cells of the *Tabula muris senis* were downloaded from: figshare.com/articles/dataset/Tabula_Muris_Senis_Data_Objects/12654728?file=23872838, and the data was further analyzed using Seurat package (version 3.1). Cells from 3- and 18-months old animals were subset as representatives of young and aged mice. In the new subset Seurat Object, clustering analysis and UMAP (Uniform Manifold Approximation and Projection) dimensionality reduction were performed using the top 3,000 variable genes and the first 20 principal components. The average expression of NSG-DR1 T cell-transferred upregulated transcripts were assigned a signature score within each cell in the Object using the “AddModuleScore” function from Seurat. The combined score of all cells from young mice was compared to cells from aged mice. Gene set enrichment analysis (GSEA) was performed as previously described (20). Briefly, normalized expression of differentially expressed genes (FDR<0.05) from all samples were organized in a tab-separated file and probed against gene sets available in the GSEA platform (https://www.gsea-msigdb.org/gsea/index.jsp). Expression profiles were compared amongst the canonical pathways from curated gene sets (https://www.gsea-msigdb.org/gsea/msigdb/collection_details.jsp#C2). Data was expressed as normalized enrichment score plus FDR with an expression heatmap depicting representative genes for each gene set. After XenofilteR deconvolution, upregulated human transcripts from T cell transferred NSG-DR1 myocardial RNA samples were selected using the XenofilteR tool (17). A list of upregulated genes that showed no counts in NSG control myocardial was used for GO and KEGG analysis in g:Profiler web-server tool.

### Methylated DNA Quantification

The percentage of 5-methylcytosine (5-mC) was determined in gDNA samples obtained from mouse myocardial tissue using the MethylFlash Global DNA Methylation ELISA Easy Kit (Epigentek). Briefly, 100 ng of gDNA samples and a standard curve (ranging from 0.1 to 5% 5-mC) were adsorbed to a 96-well assay plate for 1 h at 37°C. Wells were then washed three times with washing buffer and incubated with 50 µl of 5-mC detection complex solution, consisting of a mixture of 5-mC monoclonal antibody, signal indicator and enhancer solution for 50 min at room temperature. Following incubation, the wells were washed five times with washing buffer, and 100 µl of developing solution was added. The reaction was allowed to develop for 5 min, and then 100 µl of stop solution was added. The absorbance was read in a spectrophotometer (OD450 nm). The percentage of 5-mC in myocardial samples was estimated by interpolation of unknown values to a polynomial curve.

### Histology

Heart cryosections (14 µm) from NSG-DR1 mice following 6 weeks of CD4^+^ T cell transfer were fixed in 4% (vol/vol) formaldehyde solution in PBS and blocked with carbo-free solution (Vector Lab Inc. Burlingame, CA, USA) for 30 min and then stained with wheat germ agglutinin (WGA) FITC to stain the cardiomyocyte cell surface, followed by DAPI for nuclear counterstaining. For picrosirius red staining (PSR, Morphisto, Frankfurt am Main, Germany), heart cryosections were stained as previously described (21). For Sudan black staining, cryosections were fixed as described before and washed with 50% and 70% alcohol solution respectively. Samples were then stained for 8 min with SenTraGor^TM^ reagent (Arriani pharmaceuticals Attica, Greece) according to manufacturer’s instruction. Slides were washed twice in 50% alcohol solution and three times in PBS before proceeding to antibody staining. First, sections were blocked for mouse antibody staining with M.O.M blocking kit (Vector Lab Inc. Burlingame, CA, USA) and then stained with anti-biotin antibody clone Hyb-8 (Linaris Dossenheim, Germany) for 30 min. Following primary staining, samples were washed 3 times in PBS and stained with goat anti-mouse antibody conjugated to A555 and Phalloidin A647 for 30 min. DAPI was used at the last 5 min to stain nuclei. Samples were washed three times with PBS and mounted with Mowiol media. The fluorescence images were acquired using an epifluorescence microscope (model DFC 9000GT; Leica) coupled to a high-resolution camera (sCMOS monochrome fluorescence camera; Leica) and processed using ImageJ (NIH) software.

### Statistics

Graphs presented in the study were designed to display the group mean values (bars), the SEM and the distributions of each individual value plotted. Graphs and statistical analyses were performed using GraphPad Prism software (version 7.0d, GraphPad software). A *P* value of less than 0.05 was considered statistically significant. Differences between groups containing normally distributed data were tested using an unpaired, two-tailed *t* test for two groups or two-way ANOVA followed by multiple *t* tests for data with more than two dependent variables. The Mann-Whitney *U* test was used to compare two groups that in which data did not fit a normal distribution. The data not-fitting a normal distribution are presented as box plots showing the median and the 25^th^–75^th^ percentiles, as well as each individual point.

## Results

### Myocardial- and Immunosenescence Phenotypes Develop Synchronously

**Figure 1:**
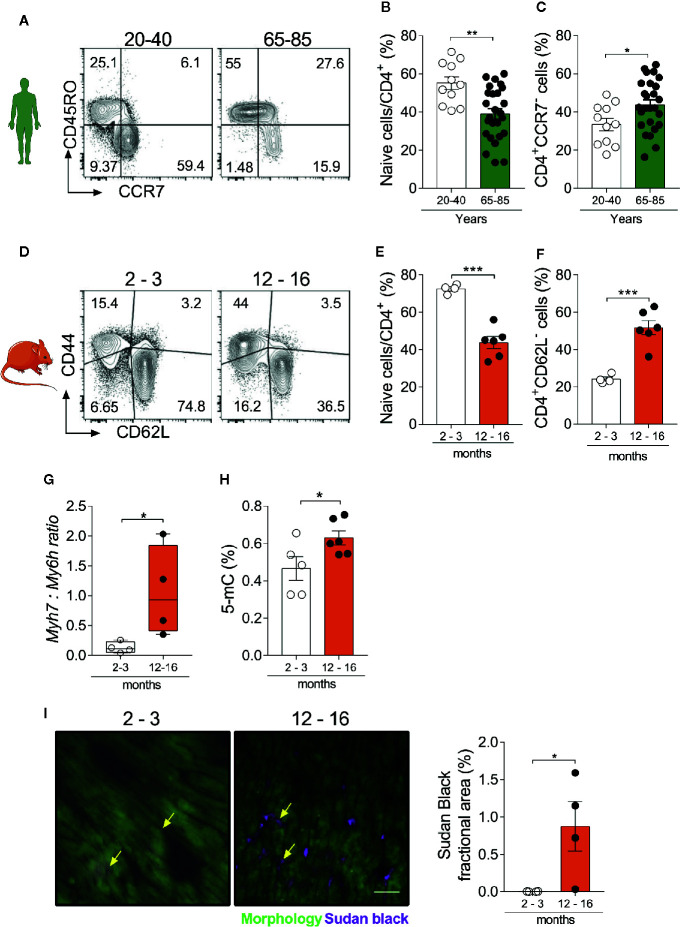
Myocardial and immunosenescence phenotypes develop synchronously. **(A)** FACS strategy depicts the frequency of human naïve CD4^+^ T cells (CD45RO^-^CCR7^+^) and the effector memory population (CD45RO^+^CCR7^-^). Aging is accompanied by a reduction in naïve CD4^+^ T cells **(B)**, while effector memory/terminally differentiated cells increase **(C)**. **(D)** FACS strategy shows the distribution of naïve (CD44^-^CD62L^+^) and effector memory (CD44^+^CD62L^-^) CD4^+^ T cells in the spleen of young (2–3 months) and aged (12–16 months) mice. The frequency of naïve CD4^+^ T cells is reduced in aging **(E)**, while effector memory and terminally differentiated cells increase **(F)**. In immunocompetent WT animals, aging is associated with and higher *myh7:myh6* expression ratio **(G)** and increased levels of heart DNA methylation **(H)**. **(I)** Immunofluorescence of lipofuscin granules and its quantification in heart tissue from young and aged mice. Yellow arrows depict lipofuscin stained areas in both groups. Scale-bar: 100 µm. The bar graphs display the group mean values (bar), the SEM and the distribution of each individual value. Statistical analysis in **(B–I)**: Two-tailed unpaired *t* test, ***P < 0.001, **P < 0.01 and *P < 0.05.

In humans, naïve T cells express the chemokine receptor CCR7 and lack the expression of activation markers such as CD45RO, while effector/memory T cells present the opposite phenotype (22). As shown in **Figure 1A** and in Supplemental **Figure 1**, the frequency of naïve CD4^+^ T cells was reduced in the elderly population (65–85 years old) compared to a younger adult population (20-40 years old) (**Figure 1B**). Similarly, effector memory and terminally differentiated CD4^+^ T cells (CCR7^-^CD45RO^+/-^) were increased in the aged group versus adults (**Figure 1C**). To translate these findings to mice, we quantified naïve (CD62L^+^CD44^-^) and effector (CD62L^-^CD44^+^) T cells in the spleen of young (2–3 months) and aged mice (12–16 months) (23) (**Figure 1D**). Our findings confirmed that shifts in naïve and effector T cell populations occur similarly between mice and humans (**Figures 1E, F**, **Supplemental Figure 2**). These age-related changes in peripheral immune cell composition have been shown to intertwine with cardiac inflammation and functional decline (11). In accordance with previous findings, we herein observed that aged hearts show a higher ratio of β:α-myosin heavy chain expression (products of the *Myh7* and *Myh6* genes respectively, **Figure 1G**), as typically seen in several pathological conditions (11, 24). In addition, cytosine methylation levels, a canonical molecular clock marker (25), were increased in the aged heart (**Figure 1H**). Moreover, Sudan black staining revealed a myocardial accumulation of lipofuscin granules in 12–16 months old hearts, especially distributed within the interstitial space (**Figure 1I**), suggesting the presence of senescent cells (26). Altogether, these data reinforce the synchronicity between immune and cardiac aging.

### Human CD4^+^ T Cells Shift Toward a Senescent-Like Phenotype When Transferred Into NSG-DR1 Mice

**Figure 2:**
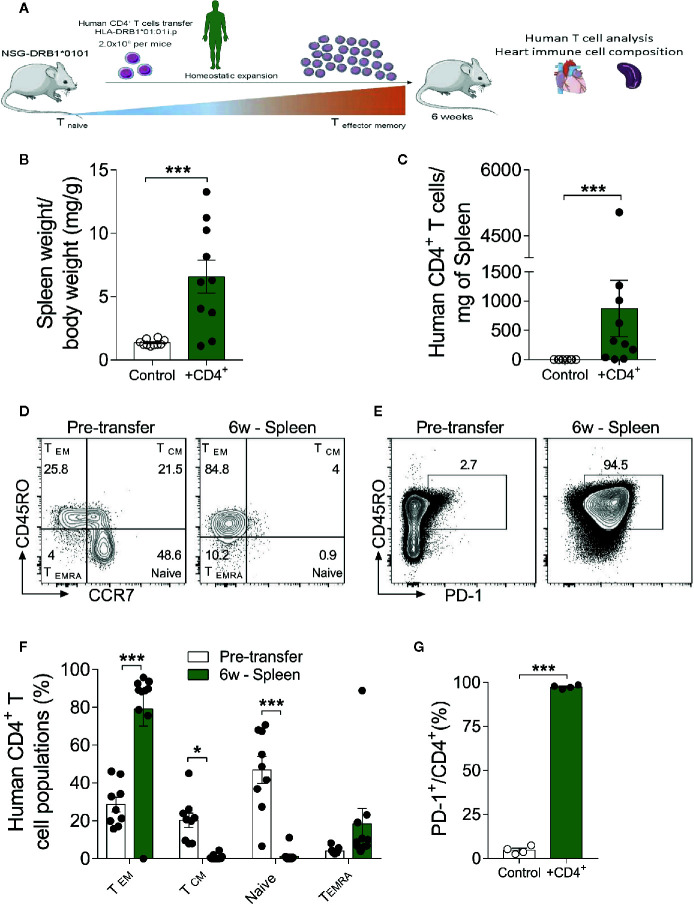
The xenograft transplantation model renders young mice harboring a senescent-like T-cell compartment. **(A)** Experimental design: Immunodeficient NSG-DR1 mice were adoptively transferred with CD4^+^ T cells purified from matching human donors, which readily undergo homeostatic expansion without causing xenograft reactions. Naïve NSG-DR1 mice were used as controls. Six weeks after transfer, heart and spleen tissue were collected for analysis. **(B)** Spleen weight to body weight ratio and number of CD4^+^ T cells per milligram of spleen **(C)** depict the T cell engraftment of NSG-DR1 mice after 6 weeks. **(D)** Flow cytometry plots illustrate the frequency of naïve (CCR7^+^CD45RO^-^) and effector memory (CCR7^-^CD45RO^+^) CD4^+^ T cells before transfer and in NSG-DR1 spleen 6 weeks afterwards. **(E)** FACS plots also illustrate the distribution PD-1^+^ T cells before and after transfer in the spleen. Control group: Naïve NSG-DR1 mice. CD4^+^ group: NSG-DR1 mice transplanted with human CD4^+^ T cells. **(F)** Bar graph shows the shift from naïve toward an effector memory phenotype following 6 weeks of engraftment in the spleen. **(G)** Increased frequency of human PD-1^+^ T cells in the spleen of NSG-DR1 mice. The bar graphs display the group mean values (bar), the SEM and the distribution of each individual value. Statistical analysis in **(B, C, G)**: two-tailed paired *t* test, ***P < 0.001, in **(F)**: two-way ANOVA followed by multiple *t* tests, ***P < 0.001 and *P < 0.05.

A CD4^+^ T cell compartment comprising inflated effector- and terminally-differentiated cells, as typically seen in aging, has been implicated in a multitude of diseases. However, it remains unclear whether these findings simply reflect the association or a causative role of immune cells in tissue senescence (27). To further dissect the contribution of a senescence-like CD4^+^ T cell compartment in a young organism context, we developed a xenograft model in which human CD4^+^ T cells from DRB1*01:01 subjects were engrafted into humanized immunodeficient NSG-DR1 mice expressing a matching human HLA (detailed in methods section). After a period of 6 weeks of T-cell expansion, the heart and spleens of recipients were harvested for downstream analysis (**Figure 2A**). Successful engraftment was demonstrated by the higher spleen to body weight ratio in transferred mice, together with the detection of human CD4^+^ T cells in the recipients’ spleens (**Figures 2B, C**). No alterations in animal body weight were observed over a 6-week period, indicating a lack of xenograft reaction (**Supplemental Figure 3A**).

The phenotype of human CD4^+^ T cells was analyzed by flow cytometry before adoptive transfer and after engraftment conditions as previously described (28). After gating on live single cell CD4^+^ events, the major T helper cell subsets were defined as naïve (CD45RO^-^CCR7^+^), effector-memory (CD45RO^+^ CCR7^-^), T central memory (CD45RO^+^CCR7^+^), and terminally differentiated (CD45RO^-^CCR7^-^) (**Figure 2D**). Moreover, exhausted T-cells were defined based on the surface expression of PD-1 (**Figure 2E**) (29), whereas regulatory CD4^+^ T cells were defined CD4^+^FoxP3^+^ (**Supplemental Figure 3C**) (30). As shown in **Figures 2D–G** and **Supplemental Figure 3B**, upon adoptive transfer into NSG-DR1 mice, CD4^+^ T cell exhibited a stark shift toward effector-memory/terminally-differentiated and exhausted phenotypes in parallel with a significant decline in the naïve and regulatory compartments. This effect was not dependent on CD4^+^ T cell donor’s age (**Supplemental Table II**). This data confirm that we have successfully generated an experimental model able to recapitulate the hallmarks of T-cell senescence in young mice, offering a unique opportunity to decompose the causality of cardio-immune aging processes.

### Human CD4^+^ T Cell Engraftment in NSG-DR1 Mice Favors Cardiac Leukocyte Accumulation

**Figure 3:**
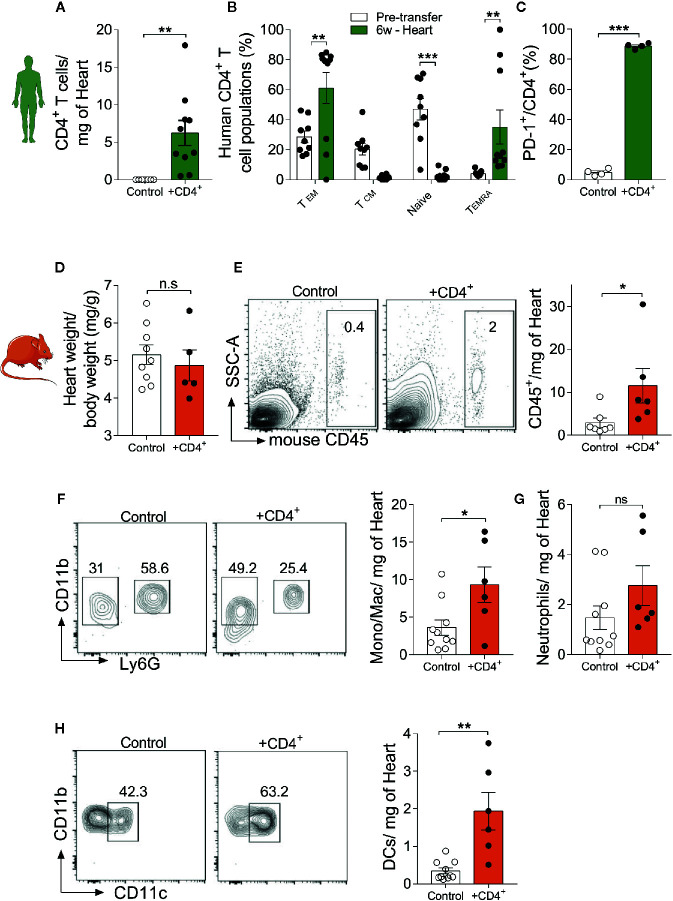
Young mice harboring a senescent-like CD4^+^ T-cell compartment show signs of myocardial inflammaging. Myocardial infiltration of transferred T-cells and its impact on baseline tissue inflammation. **(A)** Human CD4^+^ T cells were detected in the myocardium of NSG-DR1 mice 6 weeks after engraftment. **(B)** CD4^+^ T cells found in the myocardium mainly presented a terminally differentiated phenotype, classified as CCR7^-^CR45RO^-^, while naïve and central memory phenotypes were largely reduced after transfer. **(C)** Increased frequency of heart PD-1^+^ T cells versus pretransfer values. **(D)** Heart weight to body weight was measured in control and CD4^+^ T cell-transferred NSG-DR1 mice. **(E)** FACS plots depict the frequency of heart mouse leukocytes (live CD45^+^) in control and adoptively transferred mice. The bar graph shows the number of heart leukocytes normalized per tissue weight. **(F)** Gating strategy for cardiac monocytes/macrophages (CD11b^+^Ly6G^-^) and neutrophils (CD11b^+^Ly6G^+^). The bar graph illustrates the number of monocytes/macrophages and neutrophils **(G)** in control and CD4^+^ T cell-transferred mice per milligram of heart. **(H)** Cardiac dendritic cells were gated as Ly6G^-^CD11b^+^/CD11b^+^CD11c^+^ events. Control group: Naïve NSG-DR1 mice. CD4^+^ group: NSG-DR1 mice transplanted with human CD4^+^ T cells. The bar graphs display the group mean values, the SEM and the distribution of each individual value. Green bars represent parameters measured on human CD4^+^ T cells, and orange bars represent mouse endogenous leukocyte parameters. Statistical analysis in **(B)**: two-way ANOVA followed by multiple *t* tests, ***P < 0.001 and **P < 0.01, in all other panels: two-tailed unpaired *t* test, **P < 0.01 and non-significant (n.s) P>0.05. *P < 0.05.

After having observed that transferred human CD4^+^ T cells rapidly acquire an effector/terminally differentiated phenotype in NSG-DR1 mice, we sought to investigate their myocardial distribution and possible effects on the composition of other cardiac resident leukocytes. First, we observed that most transferred human CD4^+^ T cells found in cardiac tissue presented effector memory (CD45RO^+^CCR7^-^) terminally differentiated (CD450^-^CCR7^-^) and exhausted (PD-1^+^) phenotypes (**Figures 3A–C**). No differences were detected in the Treg distribution in heart tissue when compared to pretransfer frequencies (**Supplemental Figure 4A**). Moreover, cardiac infiltrating CD4^+^ T cells showed increased production of TNF and IL-13 following *in vitro* stimulation, compared to pre-transfer values and to splenic cells (**Supplemental Figure 4B-D**). The myocardial accumulation of transferred CD4^+^ T cells did not result in changes from heart to body weight ratios (**Figure 3D**), but promoted a mild infiltration of other murine inflammatory cells (**Figure 3E**), including monocytes/macrophages (Ly6G^-^CD11b^+^, **Figure 3F**) and dendritic cells (CD11b^+^ CD11c^+^ Ly6G^-^, **Figure 3H**), but not neutrophils (CD11b^+^Ly6G^+^, **Figure 3G**).

### Expanded CD4^+^ T Cells Promote Myocardial Inflammation and Stress Response

**Figure 4:**
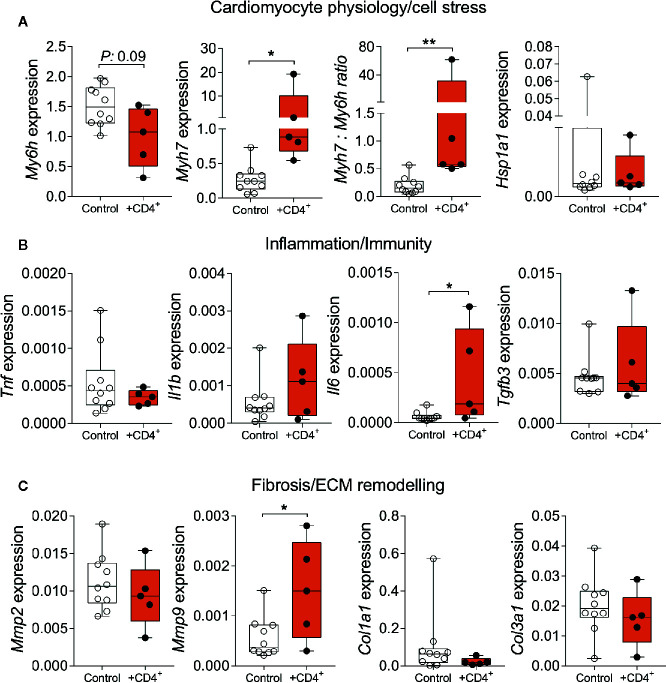
Senescent-like CD4^+^ T cells promote alterations in myocardial gene expression compatible with physiological aging. Relative myocardial mRNA levels of transcripts related to cardiomyocyte stress **(A)**, inflammation **(B)**, and extracellular remodeling **(C)**. Control group: Naïve NSG-DR1 mice. CD4^+^ group: NSG-DR1 mice transplanted with human CD4^+^ T cells. The bar graphs display the group mean values, the SEM and the distribution of each individual value. Statistical tests: Mann-Whitney. **P < 0.01 and * *P* < 0.05.

To further assess the impact of a senescent CD4^+^ T cell compartment on myocardial inflammation and the stress response, we analyzed the expression of genes that reflect features of cardiomyocyte physiology/stress response, inflammation/immunity and extracellular matrix remodeling/fibrosis (31–35). We observed a significant increase in beta myosin heavy chain 7 expression (*Myh7*) in heart tissue from T cell-transferred NSG-DR1 mice, paralleled by a reduction in alpha myosin heavy chain (*Myh6*) expression, similar to physiological aging (**Figure 4A**). In addition, T cell-transferred NSG-DR1 mice displayed an increased myocardial *Il6* expression, a proinflammatory cytokine associated with left ventricular dysfunction and a heart failure predictor in humans (32) (**Figure 4B**). Moreover, the expression levels of *Mmp9*, which can be produced by cardiomyocytes, neutrophils and monocytes under inflammatory conditions, was also found to be increased in T cell-transferred hearts (**Figure 4C**). Alongside with increased cardiac leukocyte numbers, these findings suggest that a senescent-like CD4^+^ T cell compartment is sufficient to promote mild myocardial inflammation compatible with the concept of inflammaging. No differences between groups were observed concerning the myocardial expression levels of *Hsp1a1*, *Il1b*, *Tnf, Tgfb3, Col1a1*, and *Col3a1*. Other canonical markers of cellular senescence (e.g., CpG methylation and accumulation of lipofuscin granules) and myocardial aging (e.g., hypertrophy and fibrosis) were not affected by the adoptive T-cell transfer within the short period herein analyzed (**Supplemental Figure 5**).

### Transcriptome Analysis Reveal an Inflammaging Phenotype in the Heart of CD4^+^ T Cell Transferred NSG Mice

**Figure 5:**
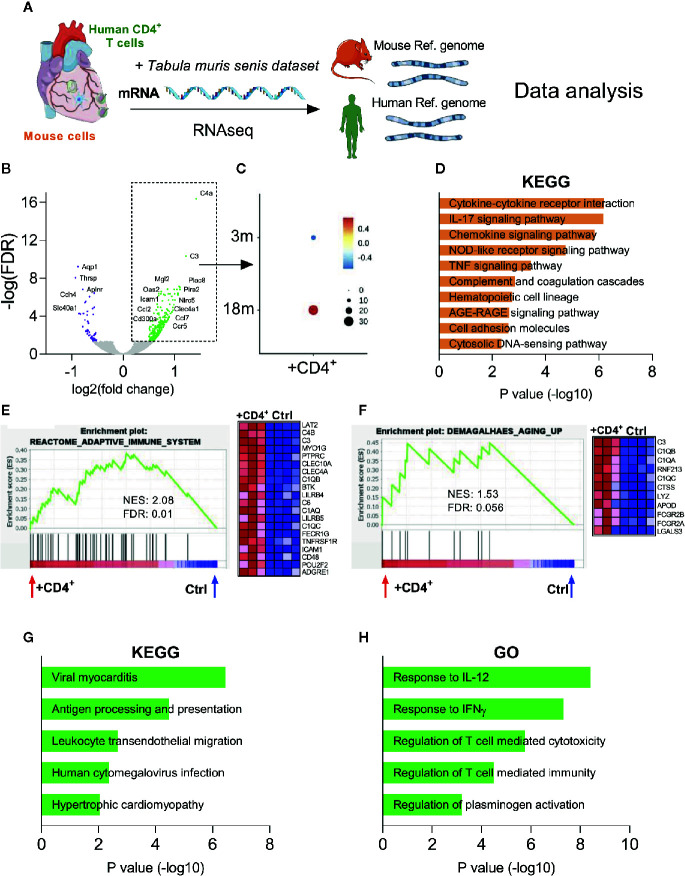
Transcriptome analysis reveal an inflammaging phenotype in the heart of CD4^+^ T cell transferred NSG mice. **(A)** Myocardial mRNA extracted from control and T cell transferred NSG-DR1 mice was employed in RNA-seq analysis. Transcripts were aligned against mouse genome and human genes were selected using the XenofilteR tool and further integrated with other publicly available datasets. **(B)** Volcano plots comparing the gene expression levels in myocardial tissue of T cell transferred NSG-DR1 mice versus control. The upregulated and downregulated genes (± 0.5–1.5 log2 fold, FDR<0.05) are highlighted in green and purple respectively. **(C)** The myocardial upregulated gene set identified in **(B)** was then probed in other myocardial aging datasets publicly available [*Tabula muris senis* (19)]. A score of the average expression levels (colored scale) and fraction of cells expressing the signature (circle sizes) was analyzed at single-cell level on myocardial samples obtained from 3- and 18-months old mice (3M and 18M respectively). **(D)** KEGG analysis of upregulated mouse transcripts from T cell transferred NSG-DR1 hearts. The bars represent the adjusted *P* value (Fisher’s exact test). **(E, F)** GSEA analysis of “reactome adaptive immune system” and “Demagalhaes aging up” (36) in the myocardium of control and T cell transferred NSG-DR1 mice. Normalized enrichment score (NES) and FDR are depicted in the graphs. The heatmaps illustrate the genes differently expressed at each gene set. **(G, H)** KEGG and gene ontology (GO) analysis of upregulated human transcripts found in the myocardium of T cell transferred NSG-DR1 mice. The bars represent the adjusted *P* value (Fisher’s exact test). Data from control (n: 4) and CD4^+^ T cell transferred (n: 3) were acquired from two independent experiments.

To gain mechanistic insight into the effects of terminally differentiated T cells on the heart inflammaging status, we performed bulk RNA sequencing of myocardial tissues from T cell transferred and control NSG-DR1 mice (**Figure 5A**). The major advantage of this approach is that the sequenced reads can be then aligned against the mouse and human reference genomes, meaning that it is possible to discriminate what transcripts were expressed by the murine myocardial cells or by the transferred human cells. As depicted in **Figure 5B**, hearts from T-cell-transferred mice upregulated a unique set of murine genes involved in complement pathway (*C4a, C3*), immune cell trafficking (*Icam1, Ccl2, Ccl7, Ccr5*), and immune inhibitory response (*CD300a, Clec4a1*). To investigate whether this transcription signature could be also upregulated in the heart during physiological aging, we compared it against cardiac single-cell transcriptomic data available from the *Tabula muris senis* consortium (19). The *Tabula muris senis* is a bold initiative that provides an unprecedented and comprehensive analyses of age-related molecular signatures in mice. It includes bulk and single-cell-sequencing RNA atlases covering 23 tissues and organs throughout the mouse lifespan, offering a unique resource to be compared with our own sequencing findings. The analysis revealed that the myocardial gene set found to be upregulated in young NSG mice harboring an expanding T-cell compartment resembles the signature observed in aging hearts (**Figure 5C**).

Unsupervised enrichment pathway analysis and Gene set enrichment analysis (GSEA) further revealed a T-cell-triggered upregulation of pathways related to myocardial inflammation (e.g., Cytokine-cytokine receptor interaction, IL-17 and TNF signaling pathways) and canonical aging pathways such as advanced glycation end products signaling (AGE/RAGE) (**Figures 5D, E**). Furthermore, the myocardial gene expression signature found in T-cell-transferred NSG-DR1 mice overlapped with an conserved age-related gene set described in several tissues of senescent mice, rats and humans (**Figure 5F**) (36). Lastly, we deconvoluted the mouse and the human transcripts found in T-cell-transferred NSG-DR1 hearts using the XenofilteR tool (17). Enrichment pathway analysis indicated that human T cells infiltrating the myocardium acquired a pro-inflammatory phenotype, with a gene signature coinciding to viral myocarditis and cytomegalovirus infection processes (**Figure 5G**). Moreover, the transferred human T cells were enriched in pathways associated to canonical Th1 responses (IL-12 and IFN-γ), cytotoxicity and plasminogen activation (**Figure 5H**). The pro-inflammatory phenotype and sustained survival of expanded human T cells is also in line with TNF secretory profile observed in T-cell stimulation functional assays (**Supplemental Figure 4 C-D**) (37, 38).

## Discussion

Aging is not a disease per se; it is an unavoidable biological process. However, its pace and intensity can greatly vary between subjects, hence differentially impacting individual susceptibility to a plethora of age-related diseases. Compelling evidence has demonstrated that an overt T cell senescence profile can be associated with several age-related conditions, especially cardiovascular diseases (11–14, 39–43). However, whether the immune system fuels local inflammation due to underlying age deterioration or triggers tissue age-related changes is not completely understood. To address this question, we have developed a xenograft model in which human CD4^+^ T cells rapidly shift towards an aged-like terminally-differentiated phenotype in young NSG-DR1 hosts. The major advantage of this model is that it recapitulates several hallmarks of T cell senescence in otherwise young and healthy mice, offering therefore a unique opportunity to decompose the mechanisms of aging (44, 45).

Herein, we observed that transferred CD4^+^ T cells expanded systemically, mildly infiltrated young healthy hearts and promoted myocardial alterations that recapitulates some of the shifts typically seen in the physiological aging process, such as the recruitment of inflammatory immune cells and upregulation of pro-inflammatory genes. Most strikingly, our bulk RNA sequencing analysis identified a distinct myocardial transcriptomic signature in young NSG-DR1 mice harboring a senescent-like T-cell compartment, which matched other conserved age-related signatures reported in previous studies (19, 36). Altogether, these findings strongly suggest a causal relationship between immunosenescece and myocardial aging.

The accumulation of senescence-associated T cells (SA-T) has already been implicated in tissue dysfunction and higher morbidity in humans (2). In addition to its acute role in autoimmune diseases and allograft rejection (46), SA-T cells were also found in chronic low-grade inflammation and tissue dysfunction that developed during physiological aging. SA-T cells have been found in the visceral adipose tissue of aged mice among macrophages and B cells. Despite presenting defective TCR-mediated proliferation, SA-T cell stimulation has been reported to result in abundant production of TNF, IL-6, and osteopontin (47). Similarly, tertiary lymphoid structures enriched in PD-1^+^ CD4^+^ T cells are observed in chronic kidney disease developed in aged mice and in humans. Although the contribution of PD-1^+^ CD4^+^ T cells is not clear, global deletion of CD4^+^ T cells resulted in a dampened inflammatory response and improved kidney function (48). Infiltrating T cells expressing IFN-γ are found in aged brains in close proximity to neural stem cells and local IFN-γ signaling has been shown to impair neural stem cell proliferation, providing a possible cause for its decline during aging (49).

A recent elegant study by Desdín-Micó et al. indicated that the premature induction of T-cell senescence in young mice drives systemic inflammaging and multi-organ morbidity, being that sufficient to recapitulate some major features of aging process (50). In a previous study we had reported that the heart-draining lymph nodes of aged mice also showed a great enrichment for Th1-polarized effector CD4^+^ T cells, and that genetic models of T helper cell ablation attenuated the myocardial inflammaging (11). Most recently, Kallikourdis’s team has shown that T-cell costimulation blockade blunts the age-related myocardial functional decline (51), providing further functional evidence for a causal link between T-cell development and myocardial aging. In humans, an exaggerated accumulation of memory T cells has been widely associated to increased cardiovascular risk, heart failure progression and overall mortality (8, 12, 14). More recently, Alpert and coauthors developed a refined human immunological age score (IMM-AGE) based on longitudinal high-dimensional flow cytometry, proteomics, and transcriptomic assessments which has been shown to predict cardiovascular events and overall mortality (2).

Humanized NSG mouse strains have been used to allow a meaningful assessment of human T cell functionality in the absence of a graft versus host reaction, especially in the context of infectious disease and cancer research (52–55). On top of the standard NSG genetic makeup, the mouse strain used in the current study was further engineered to express a hybrid murine: human MHC-II (HLA-DRB1*01:01) that enables cognate antigen interactions between mouse antigen presenting cells and human CD4^+^ T cells in an *in vivo* context (56). Thus, the transferred human T-cells can be fully stimulated in this experimental setting. It has been previously reported that the adoptive transfer of CD4^+^ T cells to immunodeficient mice lacking T, B, and NK cells (15) results in rapid proliferation of donor cells marked by robust differentiation towards a memory phenotype (57, 58). Notwithstanding, none of these studies have employed this humanized adoptive cell transfer model to investigate mechanisms of aging. Despite the advantages this model; namely, a decomposition of immune vs tissue aging mechanisms in a single mouse in a translational experimental setting, this model has some important limitations. Most notably, the time-points chosen for the end-point analyses in the present study (6 weeks) might have been too short to reproduce some of the long-term low-grade shifts occurring throughout the several months of a mouse lifespan. Therefore, we could not observe structural and functional myocardial alterations, despite the clear shifts in gene expression profile. Still, the observation that terminally-differentiated T cell compartment can promote a cardiac inflammaging molecular signature in young mice without preexisting cardiac conditions raises important mechanistic insights that help dissecting some causality of myocardial aging process.

## Data Availability Statement

The original contributions presented in the study are included in the article/**Supplementary Material**. Further inquiries can be directed to the corresponding author. Sequencing data are available at NCBI GEO (http://www.ncbi.nlm.nih.gov/geo) under the accession number GSE163413.

## Ethics Statement

The studies involving human participants were reviewed and approved by ethics committee of the University of Würzburg. The patients/participants provided their written informed consent to participate in this study. The animal study was reviewed and approved by Regierung von Unterfranken.

## Author Contributions

MDG, NH, DA, MA, MS, TH performed experiments and analysed the data; MH, NH, UH recruited the human subjects and handled the human samples; MH, MDG, IS, UH, SF and GR designed the study and interpreted data; MDG and GR drafted the manuscript All authors contributed to the article and approved the submitted version..

## Funding

This work was supported by the Interdisciplinary Center for Clinical Research Würzburg (E-354 to GCR, Z-6 to TH, and clinician scientist program to MH), the German Research Foundation (DFG grant 411619907 to GR), and by the European Research Area Network – Cardiovascular Diseases (ERANET-CVD JCT2018, AIR-MI Consortium, grant 01KL1902 to GR). NH received a scholarship from the Graduate School of Life Sciences Würzburg.

## Conflict of Interest

The authors declare that the research was conducted in the absence of any commercial or financial relationships that could be construed as a potential conflict of interest.
