# Supplementary material for: Terminally Differentiated CD4+ T Cells Promote Myocardial Inflammaging
Source: Front Immunol. 2021 Feb 19;12:584538. doi: 10.3389/fimmu.2021.584538 (PMC7935504; doi:10.3389/fimmu.2021.584538)
Supplement: Supplementary file 1 [file DataSheet_1.docx]

**Supplemental Material**

**Terminally differentiated CD4^+^ T cells promote myocardial inflammaging**

**Murilo Delgobo^1,2^, Margarete Heinrichs^1,2^, Nils Hapke^1,2^, Diyaa Ashour^1,2^, Marc Appel^1,2^, Mugdha Srivastava^3^, Tobias Heckel^3^, Ioakim Spyridopoulos^4,5^, Ulrich Hofmann^1,2^, Stefan Frantz^1,2^, Gustavo Ramos^1,2 *^**

**Content:**

Supplemental Figures 1-5

Supplemental Tables I-II

**
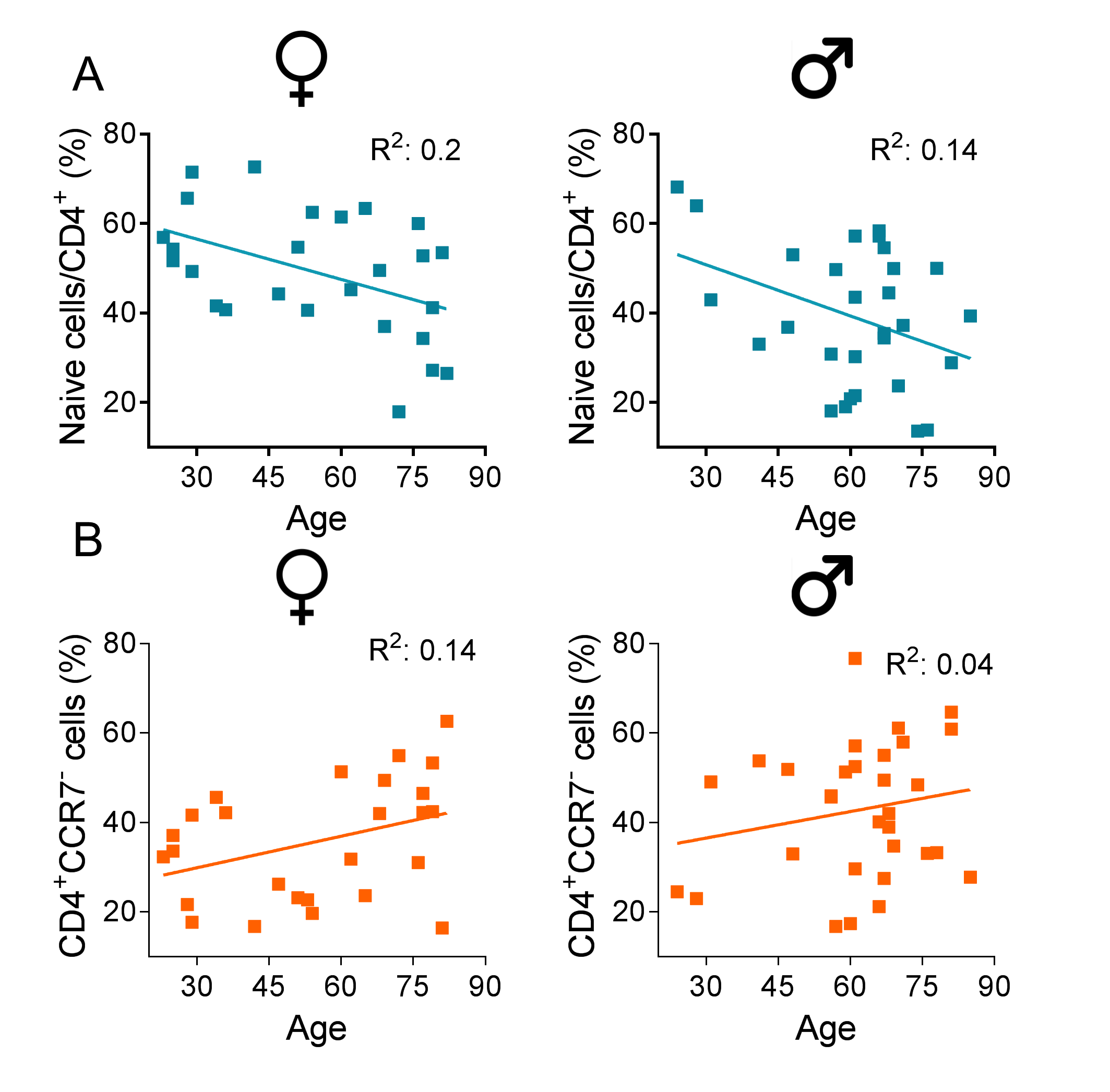
**

**Supplemental figure 1:** **Age-related shifts in the T-cell compartment in humans.** Frequency of naïve (CCR7^+^CD45RO^-^) and terminally differentiated (CCR7^-^CD45RO^-/+^) CD4^+^ T cells stratified in healthy men and women.


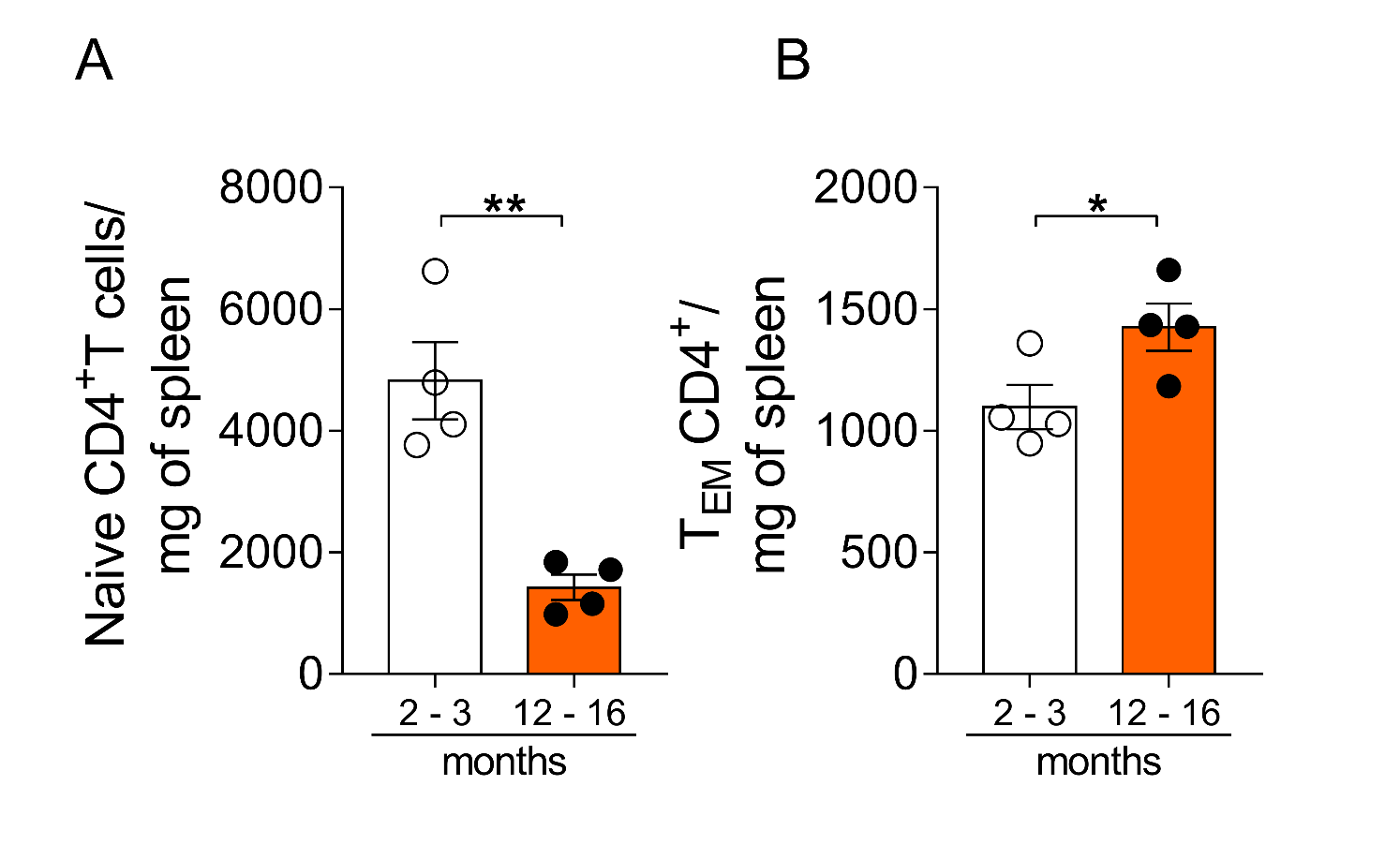


**Supplemental Figure 2:** **Age-related shifts in the T-cell compartment in mice.** **Panel A:** Naïve CD4^+^ T cells and effector memory T cell counts **(B)** per mg of spleen in young and old mice. The bar graphs display the group mean values, the SEM and the distribution of each individual value. Statistical analysis: two-tailed unpaired *t* test, ***P* < 0.01 and * *P* < 0.05.

**
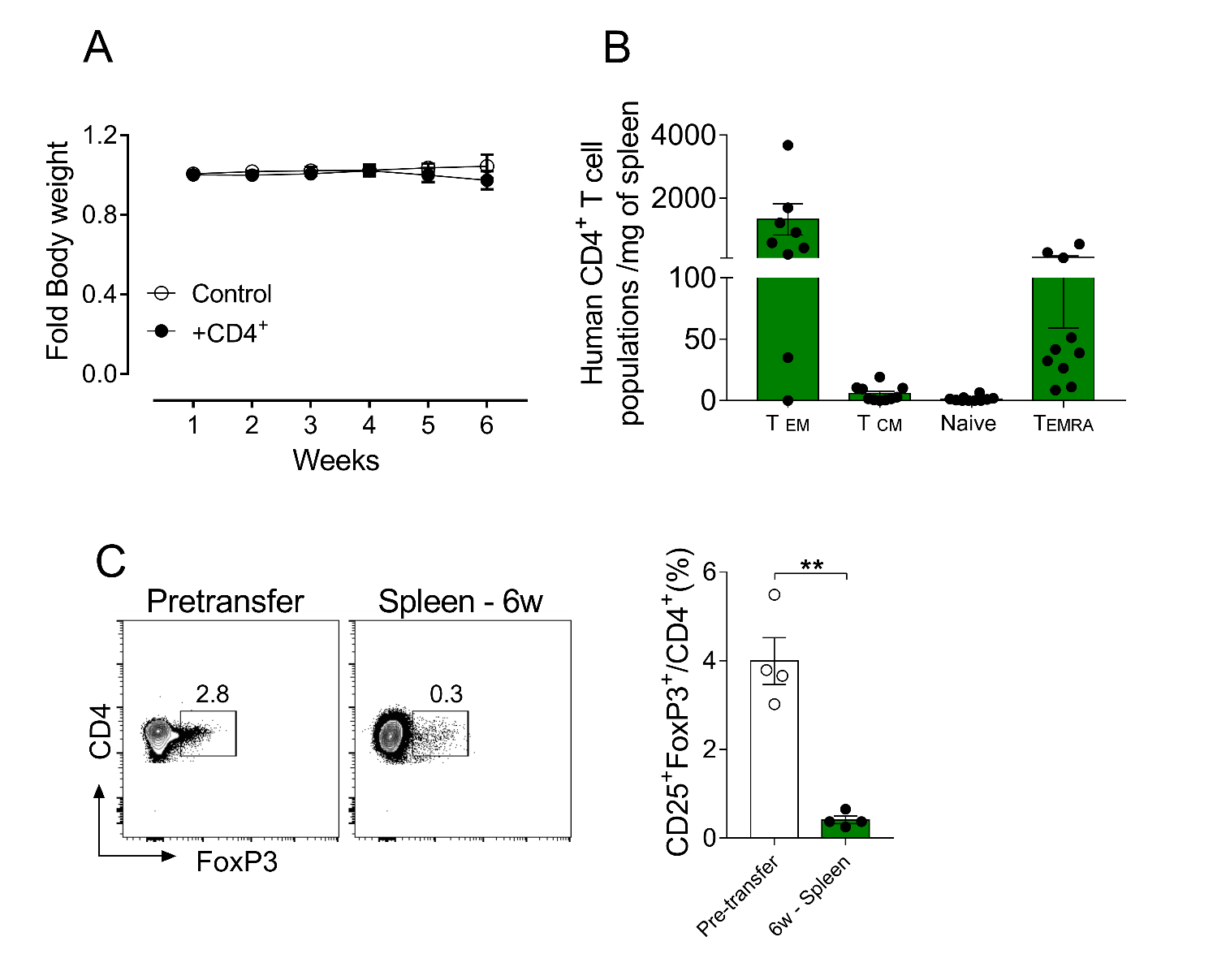
Supplemental figure 3:** **Further characterization of xenotranplantation model**. **Panel A:** Mean fold change in body weight between control and CD4^+^ T cell-transferred NSG-DR1 mice over 6 weeks. No significant differences were observed in body weight changes between groups. **Panel B:** Counts of human T cell populations normalized per mg of spleen, 6 weeks after transfer. **Panel C:** FACS plots illustrate the distribution of Tregs (CD4^+^FoxP3^+^) before and after transfer in the spleen. The bar graphs display the group mean values, the SEM and the distribution of each individual value. Statistical analysis in **C**: two-tailed unpaired *t* test, ***P* < 0.01.


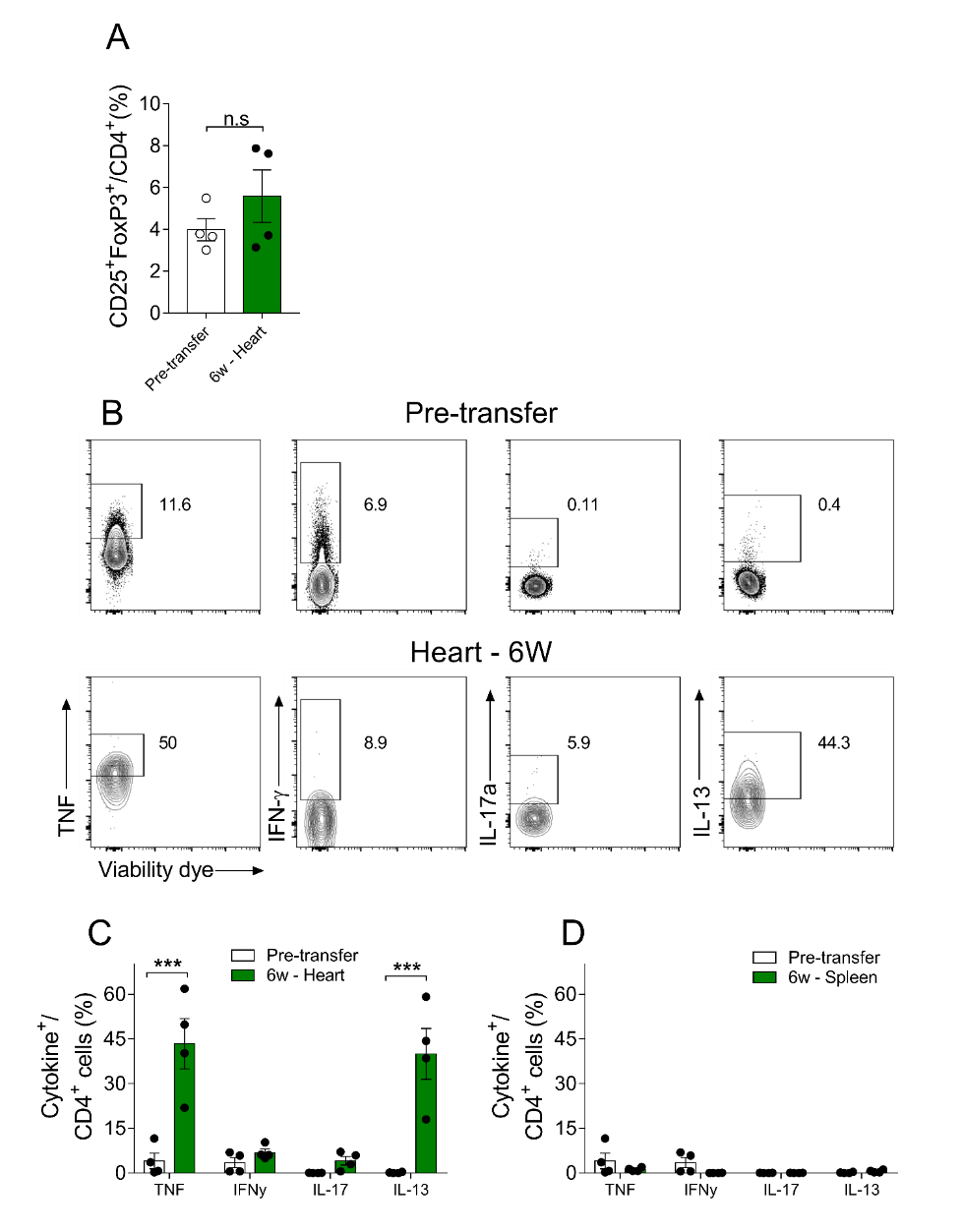


**Supplemental figure 4:** **Characterization of heart-infiltrating T-cells**. **Panel A:** Frequency of human Tregs found in the heart of NSG-DR1 mice versus pretransfer values. **Panel B:** FACS plots and graph illustrates the frequency of human CD4^+^ cytokine producing cells isolated from the heart of NSG-DR1 mice and at pretransfer condition. (C) Frequency of cytokine producing cells from heart isolated CD4^+^ T cells and spleen isolated cells (**D**) versus PBMC (pretransfer). The bar graphs display the group mean values, the SEM and the distribution of each individual value. Statistical analysis in **A**: two-tailed unpaired *t* test, *n.s* *P* > 0.05. Statistical analysis in **C** and **D**: two-way ANOVA followed by multiple *t* tests, ****P* < 0.001.


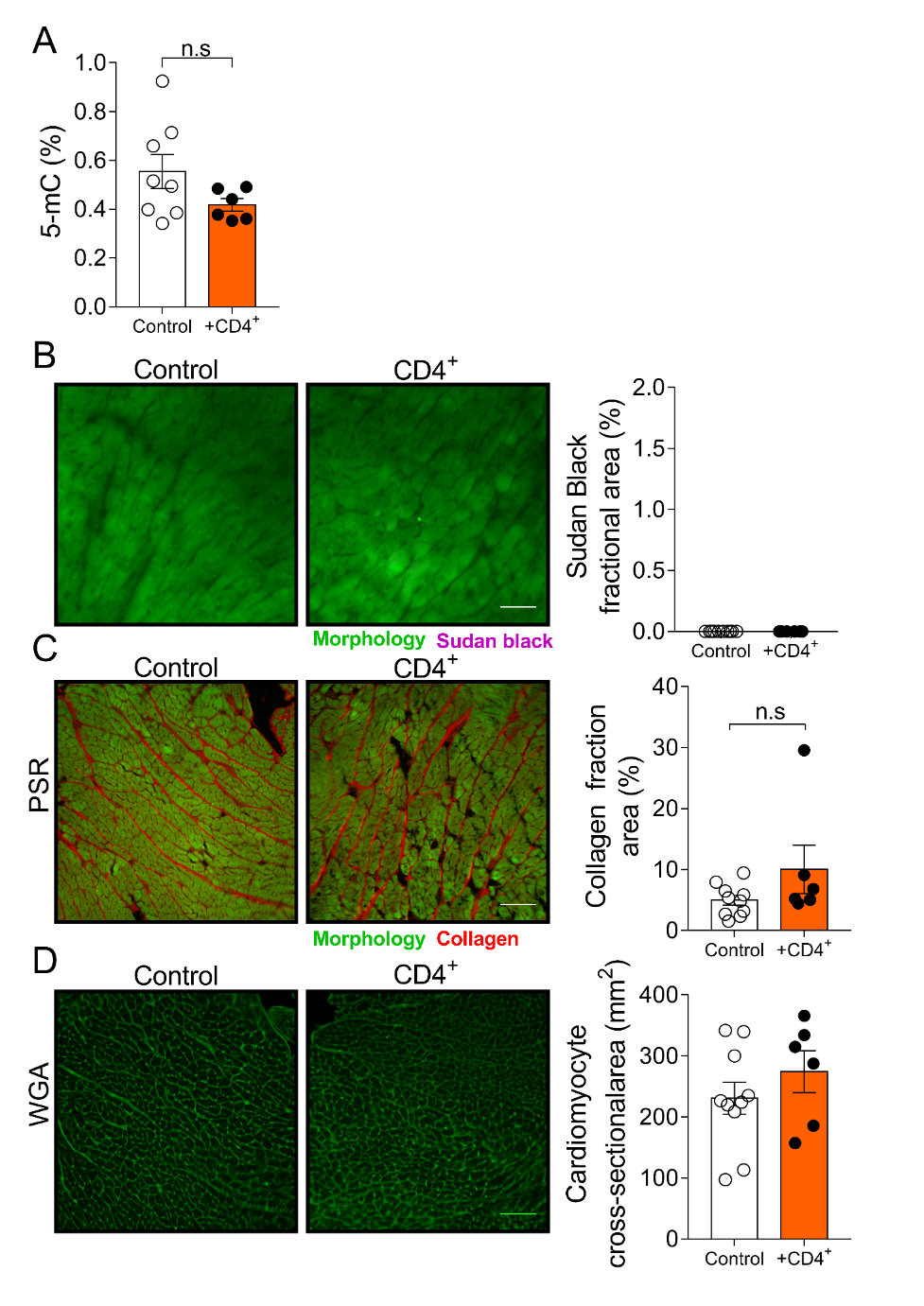


**Supplemental figure 5: Senescent-like T-cells do not alter the myocardial aging molecular clock.** Young NSG-DR1 mice harboring a terminally differentiated T-cell compartment do not show alterations in the canonical molecular markers of aging or in DNA methylation status compared to control NSG-DR1 mice (**A-D**). Scale-bar: 100µm. The bar graphs display the group mean values, the SEM and the distribution of each individual value. Statistical tests: t-test. *n.s* *P* > 0.05.

**Supplemental Table I - Frequency and average age of human subjects participating in the study.**

|  | Young subjects (20-40 yo) | Middle-aged (40-65 yo) | Elderly (65-85 yo) |
| --- | --- | --- | --- |
| Frequency of females | 0.7 | 0.35 | 0.4 |
| Age  mean ± SD | 30 (±5.3) | 56 (±5)* | 73 (±6) * |

Statistical tests: ***χ*^2^** for comparing the sex distribution and one-way ANOVA for comparing age means. * Indicates *P* <0.05 compared to the young group.

**Supplemental Table II – HLA typing of donor subjects.**

| Donor subjects | 1 | 2 | 3 | 4 | 5 | 6 | 7 |
| --- | --- | --- | --- | --- | --- | --- | --- |
| Age | 71 | 50 | 68 | 39 | 42 | 29 | 76 |
| Sex | male | female | male | male | female | female | male |
| HLA-DRB1 genotyping | 01:01 | 01:01 | 01:01 | 01:01 | 01:01 | 01:01 | 01:01 |
|  | 03:01 | 08:01 | 14:54 | 07:01 | 04:01 | 13:02 | 07:01 |
